# Race for Second Place? Explaining East-West Differences in Anti-Muslim Sentiment in Germany

**DOI:** 10.3389/fsoc.2021.735421

**Published:** 2021-11-11

**Authors:** Frank Kalter, Naika Foroutan

**Affiliations:** ^1^ Mannheim Centre for European Social Research (MZES), University of Mannheim, Mannheim, Germany; ^2^ German Center for Integration and Migration Research (DeZIM), Berlin, Germany; ^3^ Berlin Institute for Integration and Migration Research (BIM), Humboldt University of Berlin, Berlin, Germany

**Keywords:** East-Germany, ethnic rivalry, identification, islamophobia, outgroup mobility threat, recognition, social identity theory

## Abstract

It has been shown that anti-Muslim sentiment is more pronounced in East Germany than in West Germany. In this paper, we discuss existing explanations and add to them. We argue that some East Germans see themselves as a disadvantaged group in competition with other minorities, such as Muslims, for social recognition by West Germans; they are in what we call a “race for second place”. Based on social identity theory, we expect that this might be particularly true for those who explicitly self-identify as East Germans. The theoretical discussion carves out the role of “perceived non-recognition” and “outgroup mobility threat” as important concepts within the conflicts of belonging. We use unique data from the survey “Postmigrant Societies: East-Migrant Analogies” for a comprehensive empirical analysis. We find that factors related to pre-existing arguments – such as socioeconomic and demographic variables, personality traits, or contact – can capture much of the group differences in anti-Muslim sentiment, but that they do not fully apply to those who were born and still live in the East and who explicitly self-identify as East Germans. For this subgroup, perceived non-recognition adds to the empirical models and outgroup mobility threat has a stronger effect.

## Introduction and Motivation

Right-wing populism has become a political force in almost every European country, and Germany is no exception. The rise of the so-called “Alternative für Deutschland” (AfD) over the last decade is one of the most obvious indicators. The party is now^1^ represented in the European Parliament, the German Bundestag and in all 16 state parliaments. So, while there is no doubt that it is a nationwide phenomenon, there is also no doubt that the party’s success is particularly strong in the eastern part of Germany. In all five federal states that comprise the former territory of the GDR (except East Berlin), the AfD received a share of more than 20% in the last state elections, with Saxony leading the way with 27.5% (2019). In contrast, in the western federal states the maximum share is 13.1% (2018) in Hesse, while in the other states the figures are more moderate, going as low as 5.3% (2020) in Hamburg (Der Bundeswahleiter, 2021).

It is well known that populist nationalism and right-wing populism are essentially driven by aversion to racial, ethnic, religious, and sexual minorities, with immigrants and Muslims being a particularly important target (Rydgren, 2008; Golec de Zavala et al., 2017). This has also been shown to be true for AfD voting in Germany (Lengfeld and Dilger, 2018; Pickel and Yendell, 2018; Rippl and Seipel, 2018). Research has also shown that anti-immigrant and anti-Muslim attitudes are particularly strong in eastern Germany (Alba and Johnson, 2000; Zick and Küpper, 2016). Since 9/11 at the latest, antipathy, hatred and violence in Western democracies have focused strongly on Muslims, which prompts us to specifically address anti-Muslim sentiment (AMS) in this paper. They culminate not least in the rise of Pegida, the so-called “Patriotic Europeans against the Islamization of the Occident”, founded in Dresden in 2014. With the immigration of almost two million refugees to Germany in 2015 - mainly from Muslim countries such as Syria, Afghanistan, Iraq, and Iran - it has gained even more support. Although it must, again, be emphasized that anti-Muslim sentiment is by no means limited to eastern Germany, it is much more pronounced there (Zick et al., 2019: 86; Pickel and Yendell, 2018).

East-West differences in populist attitudes and behavior have received much attention in research (Kalmar, 2020; Weisskircher, 2020). Different arguments have been put forward to explain the high popularity of right-wing populism, which is associated not only with anti-immigration, but also with anti-elitist, anti-European, or anti-Western sentiment (Mudde, 2013). Its boom in the East is likely to be based on a variety of causes. These range from differences in demographic and socioeconomic composition to various contextual factors, such as economic prosperity or the proportion of ethnic minorities. An important explanatory approach also relates to the specific experiences in the GDR and the collateral damage of the transformation and reunification process (Lewicki, 2018; Küpper et al., 2019), which lead to specific psychological, political, social, and cultural dispositions and general attitudes. Empirical studies examining various indicators of right-wing populism can usually show that these arguments, taken as a whole, explain a large part of the differences between East and West, but usually cannot fully account for them.

Surprisingly, systematic accounts of how well these explanations work when looking at the specific manifestation of anti-Muslim sentiment are lacking so far. A first goal and contribution of this paper is therefore a comprehensive empirical test of whether and to what extent the available arguments, which we briefly summarize in the first subsection of the theoretical part, can also contribute to explaining East-West differences in anti-Muslim sentiment.

A second goal is to complement these explanations by developing and testing a particular strand of argumentation: Although it may sound paradoxical, it is argued that East Germans share some key experiences that are analogous to those commonly experienced by immigrants (Foroutan et al., 2019; Foroutan and Hensel, 2020). For example, East Germans continue to face economic disadvantage, underrepresentation in top positions, and prejudice. Drawing on social identity theory (Tajfel and Turner, 1979) and theories of recognition (Taylor, 1992; Lamont, 2018), we argue that anti-Muslim sentiment in East Germany can also be seen in part as a reaction to recognition gaps and can be understood as a form of “ethnic” rivalry between two minorities. East Germans compete with immigrants and Muslims when it comes to issues of representation and recognition. Pointedly, one could say that there is a kind of “race for second place”.

Within this line of reasoning, explicit self-identification, perceived non-recognition, and outgroup mobility threat become crucial concepts. In the second part of the analyses, therefore, we seek to examine whether there is evidence to support some hypotheses related to the role of these concepts in explaining anti-Muslim sentiment.

For the empirical analyses, we draw on recently collected data from the study “Postmigrant societies: east-migrant analogies” (Foroutan et al., 2020). It offers some unique features that make it particularly valuable for the purposes of this paper: it is based on a relatively large sample size with an oversampling of respondents in the eastern part of Germany. It allows East Germans to be identified not only by place of residence but also by place of birth and self-identification; this helps to disentangle some of the existing explanations. The data contain a rich repertoire of attitudinal items to capture key concepts. A specific detail of the design is that some experimental splits were implemented by randomly asking the same set of questions with respect to Muslims or with respect to East Germans, and sometimes with respect to West Germans.

## Theoretical Background, State of Research, and Expectations

### Major Frameworks and Canonical Explanations

The study of anti-Muslim attitudes in European countries, sometimes under the label of “Islamophobia”, has received increasing attention in recent years. The theoretical starting points largely overlap with research on anti-immigrant or racist attitudes in general (Strabac and Listhaug, 2008; Savelkoul et al., 2011; Helbling, 2014). Three broader frameworks are particularly influential. First, there are theories of ethnic competition or ethnic threat. These are based on the fundamental idea that the presence of an outgroup can lead to competition for scarce resources and thus be experienced as threatening by the ingroup, leading to negative attitudes toward the outgroup. The classic references here are the work of Sherif and Sherif (1969), Blalock (1967), or Blumer (1958). The core ideas have been further elaborated in different variants. Threat can be perceived objectively or only subjectively; it can be realistic or symbolic. Integrated threat theory (Stephan and Stephan, 1996; Stephan and Stephan, 2000) has been proposed as an umbrella for the influence of different forms of threat.

The second strand of argument relates to theories of intergroup contact, most famously Allport (1954) classic contact hypotheses. It stimulated an entire subfield of research on the more detailed conditions under which intergroup contact reduces negative attitudes (Pettigrew and Tropp, 2006; Hewstone and Swart, 2011).

A third traditional line of theory sees the source of negative attitudes toward certain groups in more generalized personality traits, most notably the established concept of authoritarian personality (Adorno et al., 1950) or related generalized attitudes, such as social dominance orientation (Asbrock et al., 2010) or a general fear of pluralism. In a comparative analysis using data from the 2014 European Social Survey, Pickel and Öztürk (2019) show that both realistic and symbolic threat and contact are important predictors of support for a Muslim ban in Germany as well as in the other European countries. Authoritarianism has also been shown to be a strong correlate of anti-Muslim attitudes in Germany (Pickel and Yendell, 2018: 228; Rees and Lamberty, 2019: 198).

While the descriptive fact of a general East-West difference in anti-Muslim attitudes is a stable empirical finding in diverse datasets with different indicators (Zick et al., 2019: 86; Pickel and Yendell, 2018: 226), systematic, straightforward analyses of the extent to which these explanations already explain these differences have been lacking. However, there are some findings pertaining to closely related concepts: Schmidt et al. (2019) look at attitudes toward foreigners and find that even when controlling for authoritarianism, contacts, perceived threat, and other control variables, there is still a significant East-West difference. Lengfeld and Dilger (2018) analyze the AfD vote and report a significant effect of living in East-Germany when controlling for subjective economic deprivation, satisfaction with democracy, and attitudes toward refugees. Küpper et al. (2019) focus explicitly on explaining East-West differences in right-wing populist attitudes; they summarize that several bundles of factors contribute but cannot fully explain them.

These and other studies have compiled and partially confirmed a list of explanations for the peculiarities in the East that can potentially be linked to the general theoretical frameworks and thus be used to explain the specific case of anti-Muslim sentiment: First, the sociodemographic composition of eastern Germany is somewhat different from that of western Germany. People who moved from the East to the West in the wake of reunification and its aftermath were relatively young, more educated, and overrepresented as female (Hunt 2006; Kröhnert and Vollmer, 2012) - all three factors are known to be negatively correlated with anti-immigrant attitudes. Second, the most obvious compositional effect is that the proportion of people with direct or indirect migration experience is significantly lower on average in eastern Germany: In particular, relatively few Muslims live in the eastern states (Pfündel et al., 2021: 52). This composition effect translates into a context effect, where the contact theories mentioned above take hold. Third, there is a gradient in the overall economic situation that is, despite a general trend of convergence, still visible in differences in incomes, poverty risks and other indicators (Krause, 2019). Relative economic deprivation could foster hostile attitudes according to threat mechanisms.

Another strand of argumentation relates to the socialist past and the long-term effects of specific socialization processes. This includes the widespread narrative that there would be no such thing as racism or anti-Semitism in the communist East - which, consequently, did not allow for a deeper examination of issues of racist attitudes (Küpper et al., 2019). Furthermore, communist socialization did not emphasize minority rights as an essential part of democratic values. Another important factor could be religiosity: The GDR was explicitly an atheist state and individual religiosity is still rather low in the East. However, theoretical expectations about the influence of religiosity on attitudes toward Muslims are ambiguous: while one might expect people who are religious to be more tolerant of outgroups and other religions, religiosity tends to correlate with traditional values associated with anti-immigrant attitudes (Helbling, 2014: 245). Empirical evidence on the role of religiosity on anti-Muslim attitudes is mixed (Helbling, 2014; Pickel and Yendell, 2018; Pickel and Öztürk, 2018).

### Recognition, Identification, and the Conflicts of Belonging

For a better understanding of the specifics of anti-Muslim sentiment among East Germans, it is useful to consider the fact that some of their experiences are similar to those of immigrants or ethnic minorities. Although living standards have certainly converged, the differences in wealth between West and East Germany have not completely disappeared; more than 30 years after reunification notable inequalities still exist (Krause, 2019). Moreover, East Germans continue to be poorly represented in elite social positions (Vogel and Zajak, 2020). There are still significant sensibility deficits for these empirical disparities on the part of the West and prejudices against the so-called “Ossis” (Easterners) persist (Foroutan et al., 2019). All of this leads to a persistent sense of non-recognition, which has been empirically demonstrated. For example, when asked, East Germans often responded that they feel like “second-class” citizens (Pollack, 2004). Likewise, perceptions have been articulated in recent years of feeling like “the others” (Foroutan and Hensel, 2020), strangers, or even explicitly like “immigrants” in Germany (Kubiak, 2019). At the same time, there is a growing recognition that immigrant groups, while facing similar challenges and hurdles, are increasingly experiencing success and advancement (Kalter et al., 2018; Foroutan et al., 2019). The fact that a book about eastern Germany entitled “Integrate us first” by Petra Köpping (2018), Minister of State in Saxony, made it onto the bestseller lists is a telling indication of the prevalence of this sentiment.

Thus, there is some awareness of a kind of competition between different minorities. Accordingly, existing explanations for stronger anti-Muslim sentiment in East Germany can be supplemented by approaching the research field not (only) through the lens of racism or majority vs. minority conflicts - reading East Germans as a majority and Muslims as a minority - but (also) through the lens of ethnic hierarchies and ethnic competition between minorities. Muslims, though also facing anti-Muslim resentment in the West, are a large part of the “established” West German population and were already there when East Germans “came in” in 1990. By looking at the ambivalences of belonging we try to understand the competitive part of East German Islamophobia as a contest for belonging and recognition. Thus, one way for East Germans to strengthen their self-esteem vis-à-vis the dominant West is to downgrade another comparison group and thus gain a more positive self-image.

These ideas relate to theories of recognition such as those proposed by Honneth (1996), Lamont (2018) or Taylor (1992). De Guissmé and Licata (2017) explicitly draw on them to argue that negative intergroup attitudes of minorities against each other may be due to competition for recognition which goes beyond the existing structural disadvantages. They emphasize that feelings of non-recognition are collective in nature and related to a membership in a group, and can exist even when non-recognition is not experienced individually. This is consistent with social identity theory (Tajfel and Turner, 1979), according to which individuals belong to social groups and strive for a positive self-image for themselves and their social group. Comparison of the ingroup with (an) outgroup(s) is central to this process. However, if the comparison is unfavorable, as it might be in the case of East Germans comparing themselves to West Germans, this is harmful and there are different strategies to deal with this negative affect (“positive distinction strategies”). One of them is “individual mobility”, i.e., individuals disidentify with their stigmatized ingroup and pursue individual goals which help them to assimilate to the dominant outgroup. In our case, disassociation with the ingroup would be one of the strategies for East Germans to escape their stigmatization and non-recognition in the face of disadvantage. The opposite would be “explicit self-identification (ESI)” and it is worth considering this as a key concept to analyze reactions toward negative primings.

Alternative strategies according to Tajfel and Turner (1979) are summarized as “social competition” and “social creativity”. Among the latter, one sub-option is “changing the outgroup”, i.e., the choice of a different group to be compared with.^2^ In our case, this would mean that instead of comparing the ingroup with the superior outgroup (the West), reference is made to another, apparently inferior, outgroup, the Muslims. By downgrading, stereotyping, and othering the Muslim outgroup, self-esteem and self-perception can grow (Campbell, 1967; Spivak and Harasym, 2014). But for this othering to remain consistent, the chosen outgroup, in our case the Muslims, must remain inferior. Specifically, the potential social mobility of this targeted outgroup poses a serious threat to the healed social identity. We refer to this as “outgroup mobility threat” (OMT). As a specification of ethnic threat, the concept is directly related to the threat theories mentioned above, particularly Blumer (1958) model of relative group position. While OMT can also occur on the side of the dominant majority group, it can serve as an additional sub-mechanism of social creativity for rival non-dominant groups to take action against the endangered social identity.

### Research Aims and Hypotheses

A first goal of the empirical analyses is to examine the extent to which factors related to the theoretical framework and existing empirical findings outlined above can account for the differences in anti-Muslim sentiment (AMS) between West and East in Germany. Roughly speaking, the theoretical and empirical review leads us to assume that a variety of factors contribute to this difference, including demographic, socio-economic, personality traits, and contact. As our focus is not on the already existing arguments, we treat this as a basically descriptive question and analysis, and we simply report how important the relevant variables appear in explaining the variance in AMS. Following our main theoretical arguments, our interest will rather be focused on the mechanisms surrounding the conflicts of belonging. According to the assumptions above, our general expectation is that – however well the canonical factors can already account for East-West differences – they will not be sufficient to explain the specific AMS of those who self-identify as East Germans.

*Hypothesis 1*: There will be relatively higher levels of anti-Muslim sentiments (AMS) among those in the East who explicitly self-identify (ESI) as East Germans and this difference cannot be fully explained by factors relating to pre-existing explanations (“usual suspects” being demographics, socio-economics, personality traits, and contact).

Following the theoretical arguments (see *Recognition, Identification, and the Conflicts of Belonging*) we further expect that de-identifying with the non-recognized ingroup is one possible approach to deal with the threat to social identity. Those who do not choose this coping mechanism have to resort to alternative strategies, among them “changing the outgroup”. We therefore hypothesize the following:

*Hypothesis 2*: Perceived non-recognition (PNR) is associated with greater anti-Muslim sentiment (AMS) among East-born respondents who explicitly self-identify (ESI) as East-Germans, controlling for other factors.

As OMT is a specific form of ethnic threat, we expect that:

*Hypothesis 3a*: Outgroup mobility threat (OMT) is associated with greater anti-Muslim sentiment (AMS), controlling for other factors.

However, OMT is especially threatening to those who are marginalized themselves and who rely on a favorable comparison to escape negative social identity. Accordingly, we expect that:

*Hypothesis 3b*: The relation between outgroup mobility threat (OMT) and anti-Muslim sentiment (AMS), controlling for other factors, is stronger for those East-Germans who explicitly self-identify (ESI) as East-Germans as compared to all other groups.

## Data and Methods

The data for our analyses come from the survey “Postmigrant Societies: East-Migrant Analogies” (Foroutan et al., 2020). We refer to them in the following as the “East-Mig” data for short. They consist of 7,232 telephone interviews conducted in Germany from July 2018 to January 2019 with respondents aged 14 and older.^3^ Sampling was based on a dual-frame design (landline and cell phone) (Gabler and Häder, 2009). Residents of eastern Germany, i.e., the federal states and the part of Berlin representing the territory of the former GDR, were oversampled. The dataset includes redressment weights that account for oversampling and selective nonresponse. Interviews were conducted in German. For the purposes of this study, respondents who were born abroad were excluded, reducing the unweighted sample size to *n* = 6,625.

Similar to the definition of migrant groups, the data allow us to define affiliation with West or East Germany in three general ways: by current place of residence, by place of birth, and by explicit self-identification (ESI). In our analyses, we classify respondents into a typology of five categories: 1. West-born in the West (*n* = 3,739) are respondents who were born in one of the western (old) states or West Berlin and who also currently live in the likewise defined western part of Germany. Accordingly, we distinguish 2. East-born in the West (*n* = 440) and 3. West-born in the East (*n* = 374). Among those born in the East, we further distinguish between those who 4. do not explicitly identify as East Germans (*n* = 1,308) and 5. those who do (*n* = 741). This is measured by the question “Do you feel more German or more East German?” and respondents were classified as explicitly identifying if they answered “more East German”.^4^

The central dependent variable, Anti-Muslim sentiment (AMS), is measured for all respondents with the following four items: “It should be forbidden for female Muslim students to wear a headscarf in school”^5^, “I would not mind a Muslim mayor in my community”^6^, “A Muslim woman with a headscarf should not be allowed to be part of a political TV-program”, “Exercising Muslim faith in Germany should be restricted”. Response categories were “completely agree” (=1) “rather agree” (=2) “rather disagree” (=3) and “completely disagree” (=4). The four items were rescaled so that higher values represent more negative sentiments towards Muslims. The additive index has a reliability of α = 0.70.

To capture perceived non-recognition of either Muslims or East Germans the questionnaire contains three items. The sample is randomly split into two halves, with the following questions referring either to Muslims (Split 1) or to East-Germans (Split 2): “In Germany East Germans/Muslims are treated like second-class citizens”, ‘East Germans/Muslims have to make more efforts than the rest of the population to achieve the same”, “East Germans/Muslims do not have equal access to all social positions”. Respondents could “completely disagree” (=1) “rather disagree” (=2) “rather agree” (=3) or “fully agree” (=4). Reliability of the additive index is α = 0.74 when responses for both groups are pooled (Muslim-related (Split 1) only: α = 0.69; East-German-related (Split 2) only: α = 0.75.).

The same split is implemented to capture outgroup mobility threat (OMT) by East Germans or Muslims. In our analyses, we will only use the responses that refer to Muslims (Split 1). In the respective split half, the following three items were used: “I would have a bad feeling if more and more Muslims came into important leadership positions on the labor market”, “We must be careful that educational successes of Muslims are not achieved at the expense of educational opportunities for the rest of the population”, “I’m afraid that the better off Muslims are, the more demands they make”. Here, response categories were “fully agree” (=1) “rather agree” (=2) “rather disagree” (=3) and “completely disagree” (=4) The four items were rescaled so that higher values represent more threat. The reliability coefficient is α = 0.83.

Next to these variables of key interest, we control for several independent variables in our analyses. Among them are basic socio-economic characteristics, such as gender (female = 1, male = 0), age (in years divided by 10) and migration background (1 = father or mother foreign-born, 0 = father and mother born in Germany).^7^ Education is measured in terms of the highest school degree and classified into the three categories low (no degree, Hauptschule), medium (Realschule) and high (Abitur or Fachabitur). Occupational status is measured in terms of the International Socio-Economic Index (ISEI) based on Ganzeboom et al. (1992). In the multivariate analyses we divide the ISEI score by 100 to bring the effect sizes into a more comfortable range for interpretation. Subjective economic well-being is measured by the question “If you think about your own economic and financial situation, is it very good (= 4), rather good (= 3), rather bad (= 2) or very bad (= 1)?”.

Another set of independent variables relates to attitudes and beliefs regarding the key arguments in the literature (see *Major Frameworks and Canonical Explanations*). Religiosity is measured by the question “How religious would you describe yourself? Very religious, fairly religious, moderately religious, not religious at all, or anti-religious?” Answers were coded so that higher scores reflect a higher religiosity. Authoritarianism was queried with the two items “We need strong leaders so that we can live safely in society” and “Proven behaviors should not be questioned”. Respondents could “completely disagree” (=1) “rather disagree” (=2) “rather agree” (=3) or “fully agree” (=4). The correlation between the two items is r = 0.33. Basic democratic orientations were measured by asking how important or unimportant people thought certain factors were to democracy. Three items were used to form an additive 4-point scale ( α = 0.54): “that every person has the right to express his or her opinion freely”, “that undisturbed practice of religion is guaranteed”, and “that no one may be discriminated against or preferred because of his or her origin”. Plurality anxiety was captured by the two items “no more than two genders should be officially recognized“ and “marriage for all threatens my understanding of family” (1 = “completely disagree”, 2 = “rather disagree”, 3 = “rather agree”, 4 = “fully agree”; inter-item correlation: r = 0.46). Finally, contact with Muslims was measured by how often this occurs within the circle of acquaintances and how often it occurs in the neighborhood (1 = “never”, 2 = “rarely”, 3 = “sometimes”, 4 = “often”, 5 = “very often”; inter-item correlation: r = 0.47).

Table 1 summarizes the range, the mean values and the underlying number of cases for all variables used in our analysis. Because it is of central interest, the table also reports the mean values for the subgroups of the east-west typology.

**Table 1:** Descriptives (weighted means or mean shares) of variables used

	Min	Max	Valid number of cases	Mean Total	Means, East-West typology				
					West-born in West	East-born in West	West-born in East	East-born in East, no ESI	East-born in East with ESI
Key concepts									
AMS	1	4	6,615	2.02	1.95	2.12	2.05	2.25	2.53
PNR									
Muslims	1	4	3,208	2.36	2.37	2.37	2.19	2.29	2.56
East-Germans	1	4	3,295	1.87	1.76	2.21	2.07	2.12	2.61
OMT (Muslims)	1	4	3,240	2.20	2.13	2.33	2.27	2.41	2.76
Demographics									
Age/10	1.5	9.5	6,585	5.07	5.00	4.97	4.97	5.36	5.77
Female	0	1	6,625	0.513	0.515	0.577	0.521	0.443	0.572
Migration background	0	1	6,549	0.144	0.156	0.155	0.125	0.091	0.104
Socio-economics									
Education: low	0	1	6,569	0.331	0.358	0.355	0.268	0.222	0.208
Medium	0	1	6,569	0.324	0.281	0.208	0.476	0.452	0.570
High	0	1	6,569	0.345	0.361	0.438	0.256	0.327	0.222
ISEI/100	0.116	0.890	5,449	0.478	0.485	0.469	0.451	0.478	0.448
ISEI missing	0	1	6,625	0.227	0.243	0.295	0.205	0.148	0.148
Subj. economic well-being	1	4	6,574	2.98	3.00	2.94	3.04	2.95	2.78
Attitudes and beliefs									
Religiosity	1	6	6,585	3.62	3.65	3.21	2.98	2.78	2.63
Authoritarianism	1	4	6,613	2.91	2.86	2.89	2.96	2.06	3.23
Basic democratic orientations	1	4	6,618	3.62	3.66	3.53	3.58	3.53	3.38
Plurality anxiety	1	4	6,595	1.85	1.82	1.93	1.86	1.89	2.08
Context									
Contacts Muslims	1	5	3,238	2.50	2.65	2.10	2.67	1.87	1.70
N (unweighted)				6,625	3,739	440	374	1,308	741

All means weighted; number of cases unweighted

## Results

### Testing Existing Explanations for East-West Differences in Attitudes Towards Muslims

**FIGURE 1:**
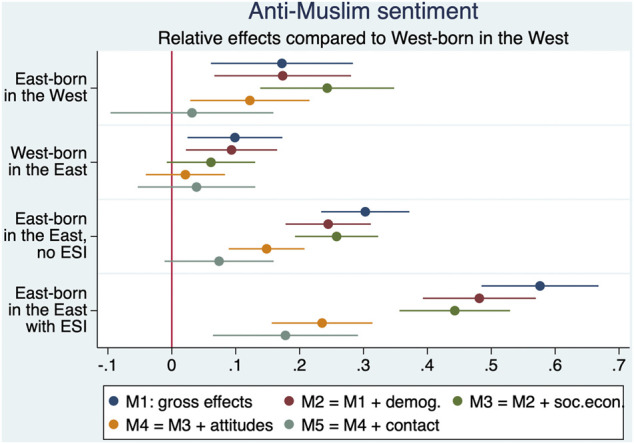
Effects of different East-West types on anti-Muslim sentiment in several OLS regressions.

We begin our empirical analyses by testing how well factors related to existing explanations can already account for East-West differences in anti-Muslim sentiment. We do this by running a series of nested OLS regressions that add stepwise sets of independent variables. The estimates for the coefficients and standard errors of the East-West typology in different models are shown in Figure 1. The full models are presented in Supplementary Table S1.

Model M2 accounts for demographic composition by controlling for age, gender, and for migration background. All three variables have a significant effect, the signs being in line with theory or previous research (see *Major Frameworks and Canonical Explanations*). Negative attitudes toward Muslims increase with age^8^ (0.12) and are lower among women (−0.05) and respondents with an immigrant background (−0.13). Comparing the effects for the East-West categories with Model M1, we find that accounting for demographic composition does not significantly change the estimates for East-born in the West and West-born in the East. However, the demographic variables do contribute to explaining the situation of the two subgroups of East-born in the East by reducing the coefficients compared to model M1 and significantly increasing the model fit with respect to the *R* ^2^.

The next model (M3) adds socioeconomic variables to the model. It includes education, ISEI value of current or last job, and subjective economic well-being.^9^ All three variables contribute significantly and in the expected direction to the explanation. Compared to respondents with low education, respondents with medium education have slightly lower (−0.09) AMS, and respondents with high education much lower (−0.39). AMS is also reduced as ISEI scores increase (one hundred points of ISEI score would change it by −0.35) and by subjective economic well-being (−0.08). However, although these socioeconomic variables contribute to the overall model fit, it is worth noting that controlling for them does not really help explain the East-West differences. On the contrary, the coefficient for East-born in the West increases (0.24) and it is interesting to see that it is now more or less at the same level as for East-born in the East without ESI (0.26). This means that the gross difference between these groups observed in model M1 can be explained by the demographic (M2) and socioeconomic (M3) selectivity of those who have migrated to the West, and this underscores the importance of considering place of birth rather than place of residence only. Similarly, West-born respondents in the East are no longer significantly (0.06) different from the reference group when demographic and socioeconomic differences are taken into account.

In the next step (model M4), we control for four key personality traits that have been surveyed in the theoretical and empirical literature (see *Major Frameworks and Canoci cal Explanations*). Religiosity has no significant effect (0.01) when controlling for the other covariates. Authoritarianism (0.18), democratic values (−0.56) and plurality anxiety (0.17) turn out to be relatively strongly correlated with the dependent variable. This is reflected in a high increase in the overall fit of the model (*R* ^2^ = 0.37) and in the fact that the effects of East-West typology are significantly reduced compared to model M3.

Model M5 extends model M4 to include contact with Muslims. Note that the questions underlying this index were asked for only half of the sample (Split 1). Therefore, model M4a reports the results from a model with the same specifications as model M4, but estimated only for respondents in Split 1. In model M5, contact with Muslims has a weak effect in the expected direction (−0.06) and slightly improves the overall fit compared to model M4a. It also has a partial mediating effect on many of the other coefficients. Most notably, controlling for contact also explains a substantial portion of the east-west type differences, as shown by the reduction in effects (except for east-born westerners) compared to M4a. In particular we find that the coefficients for three subtypes of East-West categories are close to zero and no longer significantly different from zero.

This means that the factors related to the arguments available in the literature can, by and large, explain the differences between four of the five distinguished groups. This should not be over-interpreted in a strict causal sense, as some of the variables that contribute strongly to the models, notably authoritarianism and democratic values, may suffer from endogeneity. But at least this suggests that the reasons are closely related to these personality traits. In contrast, and all the more surprisingly, the high level of anti-Muslim sentiments of those East-born respondents in the East who explicitly identify as East German cannot be fully explained, as expected. Thus, hypothesis 1 is confirmed by our analyses.

### Investigating the Peculiarities of Explicit Self-Identification

This section examines the extent to which perceived non-recognition and outgroup mobility threat contribute to explaining anti-Muslim sentiments, particularly among East-born respondents with explicit self-identification, and whether there is evidence to support hypotheses 2, 3a, and 3b.

#### The Role of Perceived Non-Recognition

**FIGURE 2:**
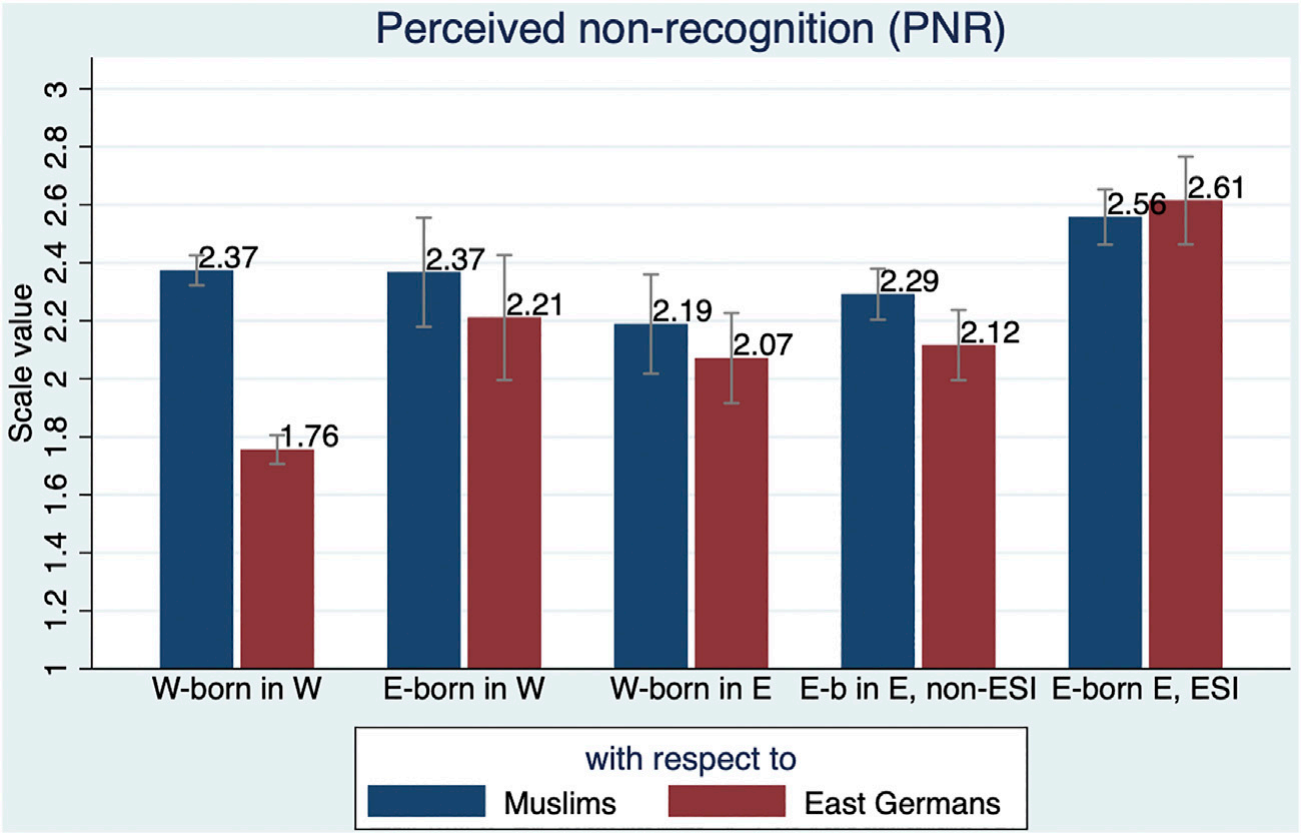
Comparison of perceived non-recognition (PNR) related to Muslims and East-Germans between East-West types.

As described in the *Data and Methods* section, a special feature of the East-Mig Survey is that a number of items were measured via an experimental random split, i.e., for half of the respondents in relation to Muslims and for the other half of the respondents in relation to East Germans. Among these items are those measuring perceived non-recognition (PNR). Figure 2 compares the mean value of the scale across the different East-West types (see also Table 1).

Among respondents living in the West, there is some perception of non-recognition with respect to Muslims; the mean score of the scale is 2.37 for both West-born and East-born respondents. The mean scores for West-born and East-born non-ESI respondents living in the East are only slightly lower (2.19 and 2.29, respectively). Somewhat surprisingly, East-born respondents with ESI tend to be even more sympathetic to the disadvantages faced by Muslims (2.56).

The picture with respect to the perception of non-recognition related to East Germans also reveals some interesting patterns: Three of the groups – East-born in the West, West-born in the East, East-born in the East non-ESI – have values slightly above 2. In contrast, West-born respondents in the West show particularly little understanding of East Germans’ difficulties, with a mean scale score of 1.76. At the other extreme, East-born respondents in the East with ESI perceive a much stronger non-recognition of East Germans (2.61). This is the only group for which the score is even higher than for the respective non-recognition with respect to Muslims. Thus, while there is a remarkable and remarkably similar understanding of Muslim disadvantage among the subgroups, there is quite a discrepancy in the assessment of whether East Germans also face similar processes of non-recognition.

To investigate the role of perceived non-recognition and test hypothesis 2, we include PNR values pertaining to East Germans in the previous regressions. Note that due to the experimental split we can do so only for half of the sample (Split 2) and we have to omit the contact to Muslims as it is asked only in the other half. This means we actually build up on the variables used in model M4. In model M6 (see Supplementary Table S2) we just include PNR as a main effect. We find that the variable weakly contributes to the model (-0.03; *p* < 0.5) and that it somewhat reduces the coefficient of East-born in East with ESI (0.21) as compared to model M4, and also the difference to the coefficient for those East-born in the East without ESI (0.17). However, we report this just for the sake of completeness and this has to be interpreted with caution: the variable PNR (related to East Germans) is an outgroup measure among West Germans, but an ingroup measure among East Germans and the described social identity mechanism leading to AMS is likely to be triggered only if there is an explicit self-identification with the ingroup. In model M7 (see also Supplementary Table S2) we therefore included PNR also as an interaction with the East-West typology.

**FIGURE 3:**
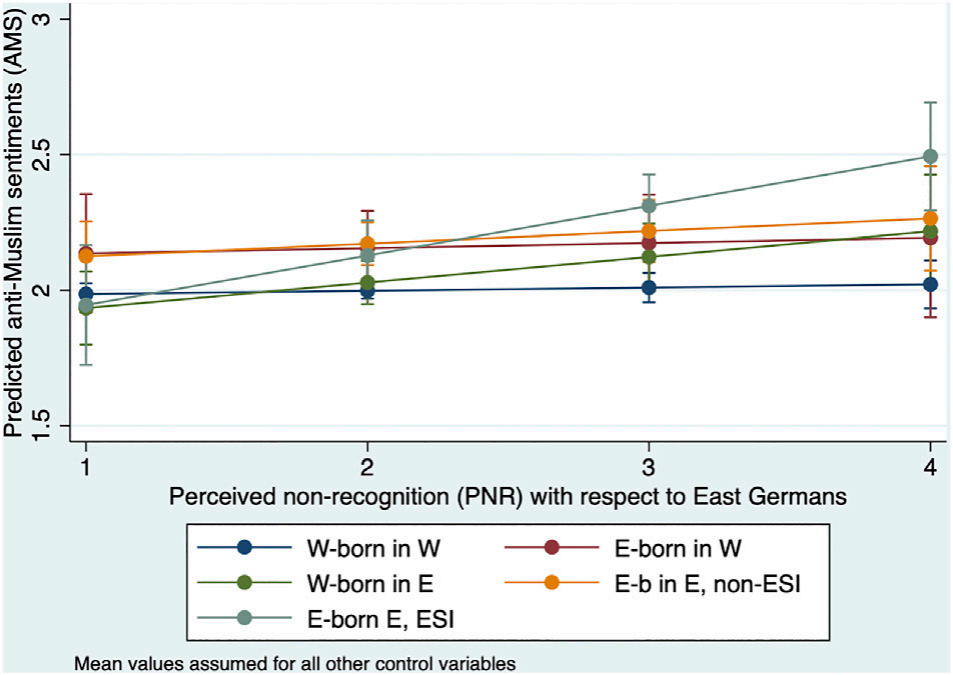
Predicted anti-Muslim sentiments (AMS) for different East-West types dependent on perceived non-recognition (PNR) with respect to East-Germans.

Figure 3 shows the predicted anti-Muslim sentiments as a function of PNR for each of the five East-West types. The values of all other control variables in model M7 are assumed to be at the means. Not surprisingly, perceived non-recognition has almost no influence on the dependent variable AMS for West-born respondents in the West. Neither does it affect AMS of East-born respondents in the West, West-born respondents in the East, or East-born respondents in the East without ESI. However, the effect is highly pronounced, as expected, for East-born respondents in the East with ESI.

The standard errors of the interaction effects in model M7 of Supplementary Table S2 show that the difference in effect size between East-born in the East with ESI and without ESI is also statistically significant at least at a 5%-level. Thus, the analysis confirms hypothesis 2.

#### The Role of Outgroup Mobility Threat

In the next step of our analyses, we examine the role of OMT and explore hypotheses 3a and 3b. As can be seen in Table 1, the overall scale mean of OMT toward Muslims is 2.20, which is lowest for West-born respondents in the West (2.13) and highest for East-born respondents with ESI in the East (2.76). As Model M8 in Supplementary Table S2 shows, OMT strongly contributes to explaining anti-Muslim sentiments, which is not surprising given the general importance of threat concepts discussed in the theoretical section. The expectation formulated in hypothesis 3a is confirmed by the results of Model M8.

Model M8 also shows that OMT further reduces the relative effect of East-born in the East with ESI (0.11) as compared to model M5 (0.18). However, although OMT is strongly correlated to AMS, the coefficient is still significantly different from zero on a 5%-level, so OMT cannot fully explain the puzzle.

**FIGURE 4:**
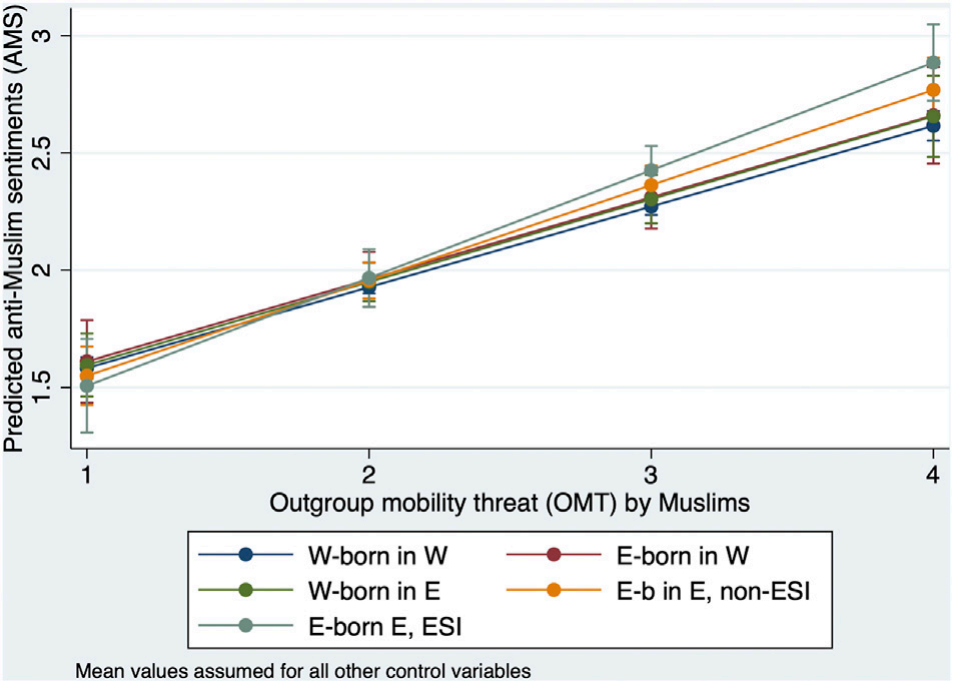
Predicted anti-Muslim sentiments for different East-West-types dependent on outgroup mobility threat (OMT) by Muslims.

As hypothesis 3b above states, not only do we expect OMT to be higher for East-born with ESI, but we also expect OMT to have a stronger effect on AMS in this group. This is confirmed by the results of model M9 in Supplementary Table S2, in which we also introduced interaction effects of OMT with the East-West typology. They show that OMT is highest in the group of East-born respondents with ESI. Figure 4 shows the predictions resulting from the estimated main and interaction effects when all other variables are set to their mean values.

We find that the effect of OMT on anti-Muslim sentiments is indeed stronger for East-born in the East with ESI than for the other groups. However, the difference in effect size is not significant when compared to East-born in the East without ESI, but it is significant at least at a 5% level when compared to the other three groups. Thus, hypothesis 3b can be partially, but not fully, confirmed.

## Conclusion and Discussion

In this paper, we have been able to show that existing explanations based on general theoretical frameworks and on available empirical evidence on East German specificities explain a large part of the empirical East-West differences in anti-Muslim sentiment (AMS). The East-Mig survey data (Foroutan et al., 2020) provide a rich repertoire of factors that have been used in previous research and can be controlled for simultaneously. The data also allow us to define the concept of “being East German” in different ways. Upon closer examination, we find that the high AMS scores of the subgroup of East-born respondents living in the East who have an explicit self-identification (ESI) as East Germans cannot yet be adequately explained.

To understand this better, we started with the fact that East Germans to some extent have similar experiences and face similar stereotypes as Muslims. We drew on social identity theory (Tajfel and Turner, 1979) and theories of recognition (Taylor, 1992; Honneth, 1996; Lamont, 2018) and discussed whether perceived non-recognition (PNR) on the part of East Germans and some sort of competition with Muslims for recognition could help explain the relatively greater anti-Muslim attitudes (AMS). The survey design provides an interesting insight into perceptions of non-recognition (PNR), as the underlying items were experimentally manipulated to measure analogous perceptions with respect to either East Germans or Muslims. It turned out that respondents in both East and West are quite aware of the disadvantages faced by Muslims in general, but that West-born respondents living in the West of Germany do not perceive these disadvantages when it comes to East Germans, whereas East-born respondents and West-born respondents living in the East do so much more strongly. East-born respondents in the East with ESI perceive themselves to be disadvantaged to a slightly higher degree than the Muslim population.

We were able to show that this sense of non-recognition is indeed related to higher levels of anti-Muslim attitudes among East-born respondents in the East with ESI. Among the other groups of respondents, however, not only is there less perceived non-recognition (PNR), but PNR (related to East Germans) does not contribute very much to explain AMS. This interaction is consistent with the expectations derived from social identity theory. It supports our main argument that some East Germans, especially those who identify strongly with the East, may see themselves as a minority or disadvantaged group competing with Muslims for recognition by the dominant group of West Germans. As a result, comparison with this outgroup serves as a strategy to achieve self-esteem associated with group membership.

We also argued that the potential social mobility of the targeted outgroup should be particularly undesirable. Accordingly, we identified outgroup mobility threat (OMT) as another important concept in this process. OMT is a strong predictor of AMS for all respondents, but it is extremely pronounced among respondents born in the East, especially those who explicitly identify as East Germans. Here, OMT is also more strongly associated with AMS. This is further evidence that the sense of competition, in this case the threat of perceived mobility, plays a notable role in understanding the especially strong negative attitudes in this group. However, the differences with the other groups are more nuanced and not as clear as in the PNR case. Although OMT contributes to explaining the difference, it alone cannot tell the whole story for those who self-identify as East Germans. As the results for PNR have suggested, it appears to be the competition for recognition that makes this group truly unique.

While all of this represents an unprecedented empirical contribution to research on anti-Muslim sentiment and to the understanding of right-wing populism, particularly in East Germany, the study must be viewed as only a first step in testing the hypothesized mechanisms, as it is fraught with limitations: There is, of course, the problem of endogeneity; in the absence of longitudinal data or useful instrumental variables for anti-Muslim sentiment and perceived non-recognition, there is the question of causality between the central concepts of ESI, PNR, and AMS. Indeed, hostility toward the outgroup can also be seen as a driver of ingroup identity processes and feelings of non-recognition. Causalities between PNR and ESI can also point in both directions. Disentangling these causalities more precisely is a task and challenge for future research.

Furthermore, according to social identity theory, as briefly mentioned above, there are additional coping strategies, including comparing on a “new dimension” (Tajfel and Turner, 1979: 43). This means questioning the superiority of the dominant outgroup by emphasizing criteria that seem more advantageous to the ingroup. In public discourse, for example, it can be observed that some of the current Eastern European regimes, even if they themselves hold anti-democratic positions, have very much adapted the narrative that they are the better or “real” democracies. They support this notion primarily through the narrative that they would protect the rest of Europe from migrant invasion and resist the Islamization of the West, thus guaranteeing European freedom (Brown, 2018). By accusing Western multicultural societies of being traitors to the people and accusing their political elite of planning a “Grande Remplacement” (Camus, 2011), they seem to positively delineate their ingroup. This pattern of argumentation has been adopted by the AfD and Pegida, explicitly even in the group names.^10^ It is thus a useful strategy to explicitly attack the dominant outgroup by questioning the value integrity of its members and claiming value superiority. However, it is not so straight-forward to conclude how this influences anti-Muslim sentiments in addition to the other mechanisms. On the one hand, it could be argued that the further belittling of the alternative marginal group, Muslims, serves to compete with the challenged dominant group by underscoring that the latter does not care enough about the most important values. On the other hand, challenging the values of the dominant group, thus “setting new dimensions”, provides an alternative to the other coping mechanisms, so one could also argue that the fact that the dominant group is targeted makes the devaluation of the other marginalized group less necessary. Exploring this and other complexities arising from alternative coping strategies is beyond the scope of this paper, but is certainly worthy of closer consideration in subsequent research.

We would like to reiterate that the arguments and mechanisms related to perceptions of nonrecognition among Eastern-born respondents that we have focused on in this paper are only one particular, complementary aspect of understanding differences in anti-Muslim attitudes. It is, as our analyses as a whole have clearly shown, only one factor among several others. Thus, the results should by no means be understood as downplaying the strong manifestations of right-wing populism in East Germany, which, as the analyses in the section *Testing Existing Explanations* have again shown, are also strongly associated with authoritarian, antipluralist, and antidemocratic attitudes. Rather, our findings on non-recognition should be understood as another piece that adds to the puzzle.

Although we have focused on anti-Muslim attitudes (AMS) as an outcome in this paper and treated them in terms of East-West differences in Germany, we believe that our arguments and results can explain much more than just this specific attitude in this specific context. AMS can be seen as a “seismograph” for measuring attitudes toward diversity, plurality, and fundamental rights in general. One could even argue that anti-Muslim sentiment can be considered a proxy for generalizable attitudes toward democracy in contemporary immigration countries. Since Muslims represent the largest cultural or religious minority in contemporary Germany, it is reasonable to expect that measures of AMS also provide an indication of the actual level of deeper democratic beliefs in Germany or other comparable countries. One could claim that “Muslim” here stands as a cipher for migration-related pluralization, and that by rejecting Muslims, other marginalized groups are also collaterally addressed. Anti-feminism, homophobia and transphobia, anti-Semitism and racism go hand-in-hand with the rise of anti-refugee, anti-immigrant and anti-Muslim attitudes. The feeling that certain social subgroups have of being a kind of minority “in their own country” and the complaints about adequate recognition are not unique to East Germans either: it is well documented that the advancement of formally marginalized groups - whether women, LGBTQ* or working-class people - can lead to status anxiety due to a possible loss of privileges in other settings (Jackson, 2006; Weis, 2006). Thus, the mechanisms highlighted in this paper could also contribute to understanding similar feelings toward other groups in other contexts by underscoring that behind them may not only be structural disadvantages and realistic threats, but also recognition gaps and a race for second place.

## Data Availability

Publicly available datasets were analyzed in this study. This data can be found here: https://doi.org/10.34882/dezim.postmig1.c.1.1.0.

## Footnotes

1 All statements about the current situation here refer to the date of July 1, 2021.

2 The other two sub-options are “setting new dimensions” and “changing the values”.

3 For a detailed technical report see Schrenker et al. (2020).

4 The response category “more as a German” was explicitly presented (read) as an alternative, but “both” was also coded as a separate category if this was the spontaneous answer. The question was asked to all respondents who were born in the East or who have at least one East-born parent. Therefore, some respondents with East-German self-identity are also in the first three categories, however the numbers are only marginal (6, 18 and 25) so that a distinction would make no sense here.

5 We were initially somewhat hesitant to include this item, because it was argued that it might measure something different, namely a tendency toward a liberal emphasis on secularism (Helbling, 2014). It is worth noting, however, that in our survey the item only correlates very weakly (r = 0.11) with the agreement to the statement “Religious symbols should be forbidden in schools” and not really (r = −0.01) negatively with the statement “I approve of Christian politicians basing their politics on their religious imprint”. On the other hand, factor analyses strongly support that the headscarf in school item loads high on a common factor of anti-Islam attitudes.

6 This item and the fourth item on restrictions to exercising Muslim faith are part of a scale that has also been used in the German ALLBUS (Breyer and Danner 2015).

7 The inclusion of respondents with a migration background in the analyses could raise some doubts, as there may be additional identification processes related to their country or culture of origin that could complicate the reasoning. But unlike first-generation migrants, who are excluded, the second generation can be assigned to the East-West typology. To be on the safe side, we ran all analyses without the second generation and found that the results are remarkably similar. Only the effects of the East-born in the West were somewhat weaker. This seems to be due to a relatively high proportion of second-generation Eastern Europeans in this group.

8 The inclusion of a quadratic term did not improve the model fit significantly.

9 Adding income categories did not improve the model fit beyond these three variables significantly.

10 Pegida stands for “Patriotische Europäer gegen die Islamisierung des Abendlandes” (Patriotic Europeans against the Islamization of the Occident).
