# Supplementary material for: Race for Second Place? Explaining East-West Differences in Anti-Muslim Sentiment in Germany
Source: Front Sociol. 2021 Nov 11;6:735421. doi: 10.3389/fsoc.2021.735421 (PMC8632242; doi:10.3389/fsoc.2021.735421)
Supplement: Supplementary file 1 [file Table1.docx]

**Supplementary Table 1:** Estimates of stepwise OLS-regressions on anti-Muslim sentiment (AMS)

|  | Models | | | | | |
| --- | --- | --- | --- | --- | --- | --- |
|  | M1 | M2 | M3 | M4 | M4a | M5 |
| *East-West typology (Ref: West-born in West)* |  |  |  |  |  |  |
| - East-born in West | 0.17 *** | 0.17 ** | 0.24 *** | 0.12 * | 0.09 | 0.03 |
|  | *(0.06)* | *(0.05)* | *(0.05)* | *(0.05)* | *(0.07)* | *(0.06)* |
| - West-born in East | 0.10 ** | 0.09 * | 0.06 | 0.02 | -0.01 | 0.04 |
|  | *(0.04)* | *(0.04)* | *(0.04)* | *(0.03)* | *(0.05)* | *(0.05)* |
| - East-born in East, no ESI | 0.30 *** | 0.24 *** | 0.26 *** | 0.15 *** | 0.11 ** | 0.07 |
|  | *(0.04)* | *(0.03)* | *(0.03)* | *(0.03)* | *(0.04)* | *(0.04)* |
| - East-born in East, with ESI | 0.58 *** | 0.48 *** | 0.44 *** | 0.24 *** | 0.22 *** | 0.18 ** |
|  | *(0.05)* | *(0.05)* | *(0.04)* | *(0.04)* | *(0.06)* | *(0.06)* |
| Age (divided by 10) |  | 0.12 *** | 0.09 *** | 0.07 *** | 0.07 *** | 0.05 *** |
|  |  | *(0.01)* | *(0.01)* | *(0.01)* | *(0.01)* | *(0.01)* |
| Female |  | -0.05 * | -0.04 * | -0.01 | -0.02 | -0.03 |
|  |  | *(0.02)* | *(0.02)* | *(0.02)* | *(0.03)* | *(0.03)* |
| Migration background |  | -0.13*** | -0.13*** | -0.11*** | -0.17*** | -0.12*** |
|  |  | *(0.03)* | *(0.03)* | *(0.02)* | *(0.04)* | *(0.04)* |
| Education (Ref.: low) |  |  |  |  |  |  |
| - medium |  |  | -0.09*** | -0.04 | 0.01 | -0.02 |
|  |  |  | *(0.03)* | *(0.02)* | *(0.03)* | *(0.03)* |
| - high |  |  | -0.39*** | -0.20*** | -0.17*** | -0.17*** |
|  |  |  | *(0.03)* | *(0.03)* | *(0.04)* | *(0.04)* |
| ISEI missing |  |  | -0.20*** | -0.12*** | -0.11* | -0.12* |
|  |  |  | *(0.04)* | *(0.03)* | *(0.05)* | *(0.05)* |
| ISEI (divided by 100) |  |  | -0.35*** | -0.15** | -0.23** | -0.23** |
|  |  |  | *(0.06)* | *(0.06)* | *(0.08)* | *(0.08)* |
| Subjective economic well-being |  |  | -0.08*** | -0.05*** | -0.02 | -0.02 |
|  |  |  | *(0.01)* | *(0.01)* | *(0.02)* | *(0.02)* |
| Religiosity |  |  |  | 0.01 | -0.00 | 0.00 |
|  |  |  |  | *(0.01)* | *(0.01)* | *(0.01)* |
| Authoritarianism |  |  |  | 0.18 *** | 0.16 *** | 0.16 *** |
|  |  |  |  | *(0.01)* | *(0.02)* | *(0.02)* |
| Democratic values |  |  |  | -0.56*** | -0.57*** | -0.58*** |
|  |  |  |  | *(0.02)* | *(0.03)* | *(0.03)* |
| Plurality anxiety |  |  |  | 0.17 *** | 0.18 *** | 0.18 *** |
|  |  |  |  | *(0.01)* | *(0.01)* | *(0.01)* |
| Contact to Muslims |  |  |  |  |  | -0.06*** |
|  |  |  |  |  |  | *(0.01)* |
| Constant | 1.95 *** | 1.38 *** | 2.11 *** | 3.14 *** | 3.20 | 3.48 *** |
|  | *(0.01)* | *(0.03)* | *(0.05)* | *(0.09)* | *(0.13)* | *(0.14)* |
| Split | 1 & 2 | 1 & 2 | 1 & 2 | 1 & 2 | 1 | 1 |
| n | 6350 | 6224 | 6150 | 6088 | 2945 | 2872 |
| R^2^ | 0.03 | 0.12 | 0.19 | 0.37 | 0.35 | 0.37 |

Estimated OLS coefficients; standard errors in parentheses; * p<0.05; ** p<0.01; *** p<0.001; redressment weights used
