# Supplementary material for: Race for Second Place? Explaining East-West Differences in Anti-Muslim Sentiment in Germany
Source: Front Sociol. 2021 Nov 11;6:735421. doi: 10.3389/fsoc.2021.735421 (PMC8632242; doi:10.3389/fsoc.2021.735421)
Supplement: Supplementary file 2 [file Table2.docx]

**Supplementary Table 2:** Main and interaction effects of key theoretical variables and East-West typology on AMS

| Key variable: | PNR | | OMT | |
| --- | --- | --- | --- | --- |
| Model: | M6 | M7 | M8 | M9 |
|  |  |  |  |  |
| Main Effects East-West typology |  |  |  |  |
| - East-born in West | 0.15 * | 0.14 | 0.03 | 0.02 |
|  | *(0.07)* | *(0.18)* | *(0.06)* | *(0.14)* |
| - West-born in East | 0.03 | -0.13 | 0.02 | 0.00 |
|  | *(0.04)* | *(0.12)* | *(0.04)* | *(0.11)* |
| - East-born in East, no ESI | 0.17 *** | 0.10 | 0.05 | -0.10 |
|  | *(0.04)* | *(0.11)* | *(0.04)* | *(0.10)* |
| - East-born in East, with ESI | 0.21 *** | -0.21 | 0.11 * | -0.19 |
|  | *(0.06)* | *(0.17)* | *(0.05)* | *(0.15)* |
| *Key variable* (main effect) | 0.03 * | 0.01 | 0.36 *** | 0.34 *** |
|  | *(0.02)* | *(0.02)* | *(0.01)* | *(0.02)* |
| *Interaction effects: key variable* |  |  |  |  |
| x East-born in West |  | 0.01 |  | 0.01 |
|  |  | *(0.08)* |  | *(0.05)* |
| x West-born in East |  | 0.08 |  | 0.01 |
|  |  | *(0.05)* |  | *(0.05)* |
| x East-born in East, no ESI |  | 0.03 |  | 0.06 |
|  |  | *(0.05)* |  | *(0.04)* |
| x East-born in East, with ESI |  | 0.17 ** |  | 0.12 * |
|  |  | *(0.06)* |  | *(0.05)* |
|  |  |  |  |  |
| Other variables controlled: | see model M4 | see model M4 | see model M5 | see model M5 |
|  |  |  |  |  |
| Split | 2 | 2 | 1 | 1 |
| n | 3105 | 3105 | 2834 | 2834 |
| R^2^ | 0.39 | 0.39 | 0.48 | 0.48 |

Estimated OLS coefficients; standard errors in parentheses; * p<0.05; ** p<0.01; *** p<0.001; redressment weights used
